# Spatiotemporal expression of the putative MdtABC efflux pump of *Phtotorhabdus luminescens* occurs in a protease-dependent manner during insect infection

**DOI:** 10.1371/journal.pone.0212077

**Published:** 2019-02-14

**Authors:** Ziad Abi Khattar, Anne Lanois, Linda Hadchity, Sophie Gaudriault, Alain Givaudan

**Affiliations:** 1 Laboratory of Georesources, Geosciences and Environment, Microbiology team, Faculty of Science 2, Lebanese University, Fanar, Lebanon; 2 INRA, UMR Diversité, Génomes et Interactions Microorganismes-Insectes, Montpellier, France; 3 Université de Montpellier, UMR Diversité, Génomes et Interactions Microorganismes-Insectes, Montpellier, France; University of Toledo Health Sciences Campus, UNITED STATES

## Abstract

*Photorhabdus luminescens* is an enterobacterium establishing a mutualistic symbiosis with nematodes, that also kills insects after septicaemia and connective tissue colonization. The role of the bacterial *mdtABC* genes encoding a putative multidrug efflux system from the resistance/nodulation/cell division family was investigated. We showed that a *mdtA* mutant and the wild type had similar levels of resistance to antibiotics, antimicrobial peptides, metals, detergents and bile salts. The *mdtA* mutant was also as pathogenic as the wild-type following intrahaemocoel injection in *Locusta migratoria*, but had a slightly attenuated phenotype in *Spodoptera littoralis*. A transcriptional fusion of the *mdtA* promoter (P_*mdtA*_) and the green fluorescent protein (*gfp*) encoding gene was induced by copper in bacteria cultured *in vitro*. The P_*mdtA*_*-gfp* fusion was strongly induced within bacterial aggregates in the haematopoietic organ during late stages of infection in *L*. *migratoria*, whereas it was only weakly expressed in insect plasma throughout infection. A medium supplemented with haematopoietic organ extracts induced the P_*mdtA*_*-gfp* fusion *ex vivo*, suggesting that site-specific *mdtABC* expression resulted from insect signals from the haematopoietic organ. Finally, we showed that protease inhibitors abolished *ex vivo* activity of the P_*mdtA*_*-gfp* fusion in the presence of haematopoietic organ extracts, suggesting that proteolysis by-products play a key role in upregulating the putative MdtABC efflux pump during insect infection with *P*. *luminescens*.

## Introduction

*Photorhabdus luminescens* is a member of the family *Enterobacteriaceae*. This bacterium is highly pathogenic to many insects and maintains a mutualistic relationship in the gut of free-living entomopathogenic nematodes [1]. On reaching the insect haemocoel, nematodes regurgitate their bacterial load in the haemolymph (insect blood). Despite the efficient immune response of the insect host, *Photorhabdus* survives antimicrobial defences in the haemolymph, including circulating antimicrobial peptides (AMPs) [2, 3]. The most attractive site for early bacterial colonization outside the haemolymph is the connective tissue within the muscle layers of the mid-gut of lepidopterans and within the haematopoietic organ (HO) of *Locusta migratoria* larvae [4, 5]. The HO is a filter that captures bacteria before phagocytosis by tissue-specific phagocytes, trapping them in nodules. These nodules are composed of haemocytes and play a key role in the cellular response by surrounding aggregated bacteria [6, 7]. Bacteria growing in the haemolymph and connective tissues produce large amounts of toxins and virulence factors, including proteases, leading to insect death by lethal septicaemia within 24 to 48 hours of infection [5, 8].

Increasing interest in bacterial behaviour during infection has led to the monitoring of pathogen gene expression in response to the ever-changing environment of the infected host to improve our understanding of host–microbe interactions [9]. Host-induced genes are required for adaptation of the microbe to the new metabolic requirements of life in host tissues, and for tissue colonization and the disruption of host cellular functions and immune responses [10, 11]. Important among these factors are Resistance/Nodulation/cell Division (RND) multidrug efflux pumps that can extrude a wide range of substrates including, besides exogenous and self-produced antibiotics, damaging endogenous metabolites and toxic xenobiotics, heavy metals, organic pollutants, plant-produced compounds, quorum sensing signals, and bacterial toxins, among others [12–15]. Hence, some RND drug efflux systems are required for colonization and full virulence *in vivo* [16–20], and were recently shown to impact virulence factor production and adaptive responses via periplasmic sensor proteins in *Vibrio cholerae* [21].

The best studied RND efflux pumps are AcrAB-TolC and MdtABC-TolC in *Escherichia coli* and *Salmonella enterica* [22–25], and MexAB-OprM in *Pseudomonas aeruginosa* [26, 27]. In *E*. *coli*, the *mdtABC* (multidrug transporter ABC, formerly known as *yegMNO*) genes encode the MdtABC drug efflux system that comprises the transmembrane MdtB/MdtC heteromultimer and MdtA membrane fusion protein. This export system requires the multifunctional outer membrane channel TolC for its function, encoded by the *tolC* gene not located in the same cluster [28]. These broad-specificity transporters decrease the cellular accumulation of structurally diverse substrates some of which are also inducers of host origin such as bile salts, AMPs, plant flavonoids, and fatty acids, thereby conferring multi-drug resistance (MDR) in these bacteria [29–31]. Moreover, RND efflux pumps were shown to have a role in microbial interspecific competition and establishment of infection by protecting pathogenic bacteria against antibiotics produced by the microbial community present in the same environment [14]. In line with all these, the MdtABC efflux pump has been shown to be required for full *Salmonella* colonization of intestine and virulence in mice, as well as for full ability of the plant pathogen *Erwinia* to multiply in apple rootstock [25].

A key to understanding bacterial strategies to effectively exploit these multiple efflux pumps lies in the regulation of pump expression. The currently available data show that multidrug transporters are often expressed under precise and elaborate transcriptional control by local and global regulators [15, 32]. The expression of different RND efflux pumps could be induced by different host-produced compounds, contributing to a successful colonization of different sites within the host during the infection process [12]. In *E*. *coli* and *Salmonella*, the expression of the multidrug efflux pump MdtABC is regulated by two stress response systems, Bae and Cpx. The BaeSR and CpxARP two-component signal transduction systems respond to damage of the cell envelope; however, they differ with regard to specific inducers. BaeR is considered the primary regulator of *mdtABC* in response to a wide range of environmental stresses *in vitro*, including exposure to indole, tannins, flavonoids, tungstate, zinc, and copper that are also substrates for this pump along with bile salts derivatives, SDS and novobiocin [23, 28, 33, 34]. The overlapping substrate specificities of Mdt transporters among enterobacteria for novobiocin and bile salts suggest that these compounds may be similar to the natural substrates of these pumps such as sodium tungstate and flavonoids [35]. Pletzer and Weingart demonstrated that the expression of the *mdtABC* operon in the phytophatogen *E*. *amylovora* was induced by the plant polyphenol tannin in a BaeSR-dependent manner. They further showed that MdtABC was upregulated *in vivo* during bacterial growth *in planta* suggesting a role of this efflux pump in resistance towards antimicrobial plant compounds, such as flavonoids [36]. Similarly in *P*. *aeruginosa*, expression of the *mexAB-oprM* genes is increased in the upper layer of a biofilm upon colistin exposure so that a spatially distinct subpopulation of metabolically active cells develop tolerance to this AMP [37]. For instance, there is no data available about RND efflux pumps of entomopathogenic nematode-associated bacteria, and here we propose to monitor their expression and to assess their roles in the life cycle of *Photorhabdus luminescens* within its insect hosts.

In the present study, we identified genes from *P*. *luminescens* encoding a putative MdtABC efflux pump, which shows sequence similarity to other proven efflux pumps of enterobacteria. The *mdtA* mutant showed wild-type levels of MDR and virulence in insects. We then showed that the *mdtABC* operon was specifically induced during late stages of insect infection, within bacterial aggregate formations in the connective tissue of *L*. *migratoria* HO and that of *S*. *littoralis* midgut. Finally, we described a protease-dependent induction of the *mdtABC* promoter *ex vivo* in a HO extract-containing medium.

## Materials and methods

### Bacterial strains, plasmids and growth conditions

The bacterial strains and plasmids used in this study are listed in Table 1. *Photorhabdus luminescens* TT01 [38] was routinely grown in LB broth (DIFCO) or on nutrient agar (DIFCO) at 28°C. *Escherichia coli* XL1-Blue MRF’ (Stratagene) and S17.1 (Simon, 1984) were routinely grown in LB or on LB agar at 37°C. When required, the final concentrations of antibiotics were: 10 μg.ml^-1^ gentamicin (Gm) for strains harbouring the pBBR1-MCS5 plasmid, 30 μg.ml^-1^ Gm for strains harbouring the pJQ200KS plasmid, 20 μg.ml^-1^ kanamycin (Kan), 20 μg.ml^-1^ chloramphenicol (Cm) for *E*. *coli* and 15 μg.ml^-1^ Cm for *P*. *luminescens*.

**Table 1:** Strains and plasmids used in this study.

Strain or plasmid	Genotype and relevant characteristics	Reference or source
**Strains**		
*Photorhabdus luminescens*		
TT01	Wild type isolated from *Heterorhabditis bacteriophora* nematode TH01	[38]
Δ*mdtA*	TT01 *mdtA*::ΩCm	This study
*Escherichia coli*		
XL1-Blue MRF’	Δ*(mcrA)183*Δ*(mcrCB-hsdSMR-mrr)173 endA1 supE44 thi-1 recA1 gyrA96 relA1 lac [*F’ *proAB lacI*^*q*^*Z* Δ*M15 Tn10(Tet*^*r*^*)]*	Stratagene
S17.1	*pro* r- n- TpRSmR RP4-2-Tc::Mu::Tn*7 recA thi*	[39]
**Plasmids**		
pUC19	Amp^R^ cloning vehicle	Biolabs
pBBR1-MCS5	Gm^r^ *mob* broad-host range vector	[40]
pPROBE’-*gfp*[AAV]	Plasmid (pBBR1 replicon) containing *gfp*[AAV] gene downstream from a multiple cloning site, *kan*^*R*^ *mob*	[41]
pBB-*mdtABC*	pBBR1MCS-5 carrying *mdtABC* from TT01	This study
pJQ200 KS	Gm^r^ *sacRB mob oriV* (p15A replicon) suicide vector	S. Forst
pUC-*mdtA*-ΩCm	4.8 kb *Xba*I-*Sac*I fragment containing the 690 bp *mdtA* upstream region*-* ΩCm- the 603 bp *mdtA* downstream region, in pUC/*Xba*I-*Sac*I	This study
pJQ-*mdtA*-ΩCm	4.8 kb *Xba*I-*Sac*I fragment containing the 690 bp *mdtA* upstream region*-* ΩCm- the 603 bp *mdtA* downstream region, in pJQ200KS/*Xba*I-*Sac*I	This study
pHP45- ΩCm	AMP^r^ Cm^r^ interposon ΩCm	[42]
P_*mdtA*_- *gfp*[AAV]	pPROBE’ with *gfp*[AAV] under the control of the P*mdtABC*	This study
P_*lac*_- *gfp*[AAV]	pROBE’GFP-AAV carrying the constitutive lactose promoter (constitutively expresses *gfp*[AAV])	This study

### Construction of the *P*. *luminescens* Δ*mdtA* mutant

We created a stable chromosomal mutation in the *mdtA* gene of *P*. *luminescens* TT01 (*mdtA*_*Pl*_) by allelic exchange, by constructing a derivative of pJQ200KS carrying the upstream part of the target *mdtA* gene (690 bp extending from positions—532 to + 158 with respect to the *mdtA* translation initiation site), an ΩCm antibiotic resistance cassette [42], and the downstream part of the target gene (603 bp extending from positions—453 to + 130 with respect to the *mdtA* stop codon). TT01 genomic DNA fragments were amplified by PCR with the oligonucleotide primer pairs 5’ -GCGCTCTAGAATCATCGGAAGCCGTATCTG- 3’ / 5’ -CGCGGATCCGGGGGGTAAAGGGGAATGTCT- 3’ (upstream region) and 5’-CGCGGATCCGGTTGTTAGTGCCTGGGATCG- 3’ / 5’ -GCGCGAGCTCCCACTTCCGGTAATGCAGAC- 3’ (downstream region). The PCR products were restricted with *Xba*I/*Bam*HI and *Bam*HI/*Sac*I enzymes, the recognition sites of which are underlined. The ΩCm antibiotic resistance cassette was isolated as a 3.5 kb *Bam*HI fragment from the pHP45-ΩCm plasmid. This 3.5 kb DNA fragment was then ligated into the *Bam*HI site, between the upstream and downstream *mdtA* regions, the resulting fragment being inserted into the pUC19/*Xba*I-*Sac*I vector to generate pUC-*mdtA*-ΩCm. The full-length 4.8 kb *mdtA*-ΩCm DNA fragment was gel-purified, digested with *Xba*I and *Sac*I and inserted into the corresponding restriction sites of the pJQ200KS vector, yielding pJQ-*mdtA*-ΩCm. The pJQ200KS plasmid is a derivative of pACYC184 carrying the *sacB* gene and the *mob* site from RP4. The pJQ-*mdtA*-ΩCm plasmid was used to transform *E*. *coli* strain S17.1 and was introduced into *P*. *luminescens* TT01 by mating, as previously described [43]. Cm^R^ and Sac^R^ exconjugants were selected on 3% sucrose and LB agar supplemented with chloramphenicol. The 612 bp *mdtA* deletion and the omega insertion were checked by PCR analysis and DNA sequencing (MACROGEN, Seoul, Korea). The polar effect of *mdtA* mutation was checked by reverse transcription PCR (RT-PCR). The resulting recombinant clone was named Δ*mdtA*.

### Construction of vectors for complementation assays

The entire *mdtABC* operon of *P*. *luminescens* TT01 (*plu2774*-*plu2776*) (https://www.genoscope.cns.fr/agc/mage) was amplified by PCR from TT01 genomic DNA with the high-fidelity Herculase DNA polymerase (Stratagene) and the oligonucleotide primers 5’-GCGCCTGCAGGCCTTGCGTTTGAGAGAATG-3’ and 5’- GCGCTCTAGACCACTGATGCCGAGTTTCAT-3’, which include recognition sites for *Pst*I and *Xba*I, respectively. The resulting 7.5 kb fragment was digested with *Pst*I and *Xba*I and ligated with the pBBR1MCS-5 vectors digested with the same enzymes. *E*. *coli* XL1-Blue MRF’ was transformed with the ligation product, according to standard protocols [44]. The resulting plasmid, pBB-*mdtABC*, was checked by restriction and sequencing (MACROGEN, Seoul-Korea) and transferred into Δ*mdtA* by mating [43]. We then checked, by RT-qPCR, that the *mdtABC* operon was expressed in the complemented mutant.

### Determination of the minimum inhibitory concentrations of toxic compounds

*In vitro* susceptibility tests to determine minimum inhibitory concentrations (MICs) for wild-type *P*. *luminescens* and its derivatives were performed for a range of antimicrobial peptides (AMPs): cecropin A (Sigma), polymyxin B sulphate (Sigma), colistin methanesulphate (Sigma) and *Spodoptera frugiperda*-derived cecropin B [45]; antibiotics (Sigma): novobiocin, kanamycin, erythromycin, tetracycline, ampicillin, cefoxitin, rifampicin, ciprofloxacin, cefaclor, cefuroxime, ceftriaxone, enrofloxacin, and nalidixic acid; dyes: Bromothymol blue; metals (Sigma): copper (CuSO_4_), magnesium (MgSO_4_), and zinc (ZnSO_4_); bile-salts: sodium deoxycholate (Sigma); detergents: sodium dodecyl sulphate (Fluka), and quaternary ammoniums: Triphenyl tetrazolium chloride (Sigma), and flavonoids (Sigma): quercetin, rutin and saponin. Efflux pump inhibitors 1-(1-naphthylmethyl)-piperazine (NMP) (Chess GmbH) and Phe-Arg-β-naphthylamide dihydrochloride (PAβN) (Sigma) were used at concentrations of 50 μg.ml^-1^ and 25 μg.ml^-1^, respectively. Briefly, 96-well microtiter plates containing various concentrations of each antimicrobial agent to be tested were prepared by two-fold dilution in LB broth supplemented with gentamicin, if necessary. Wells were inoculated with 10^3^ CFU of mid-exponential growth-phase bacterial cultures. Growth was scored visually after 48 hours of incubation at 28°C. Additional *in vitro* studies of bacterial kinetics were carried out with an Infinite M200 microplate reader (TECAN) and growth was monitored by following the change in Absorbance at 600 nm (A_600nm_) at 30-minute intervals.

### Construction of transcriptional fusions with the green fluorescent protein gene *gfp*[AAV]

The P_mdtA_*-gfp*[AAV] and P_*lac*_- *gfp*[AAV] transcriptional fusions were constructed by inserting the promoter regions of the *mdtA* gene (P_*mdtA*_) and of the lactose operon (P_*lac*_) upstream from a promoter-less *gfp*[AAV] gene [46] located on a low-copy plasmid, pPROBE’GFP-AAV [46]. Briefly, the 332 bp P_*mdtA*_ corresponding to the sequence extending from positions—338 to—6 relative to the translation start site of the *mdtA* gene was amplified by PCR from *P*. *luminescens* TT01 genomic DNA with sense 5’-GCGCGAATTCCGCAGGAATATGTTCTATACACTATGG-3’ and antisense 5’-CGCGGATCCGACATTCTCTCAAACGCAAGG-3’ primers, in which the *Eco*RI and *Bam*HI recognition sites, respectively, are underlined. Similarly, the P_*lac*_ promoter (120 bp) was amplified by PCR from the bluntII-TOPO vector (Invitrogen), with sense 5’-GCTCTAGAGCGCAACGCAATTAATGTG-3’ and antisense 5’-GGGGTACCAGCTGTTTCCTGTGTGAAATTG-3’ primers, in which the *Xba*I and *Kpn*I recognition sites, respectively, are underlined. The PCR products were purified and inserted upstream from the *gfp*[AAV] gene of pPROBE’*gfp*-AAV, to generate P_*mdtA*_-*gfp*[AAV] and P_*lac*_-*gfp*[AAV]. Mating experiments were performed as previously described [43], to transfer P*mdtA*-*gfp*[AAV] and P*lac*-*gfp*[AAV]-containing plasmids into *P*. *luminescens* strain TT01, resulting in *P*. *luminescens-*P*mdtA*-*gfp*[AAV] and *P*. *luminescens*-P*lac*-*gfp*[AAV], respectively. Plasmid stability was checked in bacteria isolated from infected insects.

### *In vivo* infection assays

The Noctuidae species studied (*S*. *littoralis and S*. *frugiperda)* and locusts, *L*. *migratoria*, were reared on an artificial diet at 23°C and on grass at 30°C, respectively, with a photoperiod of 12 h. Larvae were selected and surface-sterilised with 70% (v/v) ethanol before intrahaemocoel injection. All the bacterial strains used were grown to late exponential growth phase in LB broth, washed and diluted in LB medium supplemented with 20 μg.ml^-1^ km, when necessary. We injected 20 μl each of bacterial suspension, containing 10^3^ bacteria (for *S*. *littoralis* and *S*. *frugiperda*) or 10^4^ bacteria (for *L*. *migratoria*), into groups of 20 larvae. LB medium was injected as a control into groups of 10 larvae. Treated larvae were incubated for up to 96 h, and the time of death of the insects was recorded. Bacterial concentrations, in CFU, were determined by plating dilutions on nutrient agar. Statistical analyses were performed, as previously described [43], with SPSS (SPSS, Chicago, IL), for the comparison of survival rates.

### Insect dissection and histology

We assessed the *in vivo* expression of the *gfp*[AAV] transcriptional fusions in the body cavity of the insect by dissecting, at regular time points, the larval cuticle dorsally, removing pieces of the organs of interest (the haematopoietic organ in *L*. *migratoria* and mid-gut connective tissues of *S*. *littoralis* and *S*. *frugiperda*), squashing them between the slide and cover slip and viewing them under fluorescence conditions. 10 μl of insect haemolymph were examinated at regular intervals, by fluorescence microscopy (Leica).

### *In vitro* reporter gene assays

We analysed the kinetics of gene expression *in vitro* by inoculating fresh LB containing the appropriate concentrations of kanamycin and a potential inducer to be tested, in black-sided clear bottomed 96-well plates (Greiner), with a 1/500 dilution of an overnight culture of wild-type TT01 strain carrying one of the various GFP fusions. The use of such a high dilution (1/500) ensured that GFP proteins present in the inocula were diluted out by the first cell divisions and do not interfere with the monitoring of gene expression. Plates were incubated for 48 h at 28°C, with orbital shaking, in an Infinite M200 microplate reader (Tecan). A_600nm_ and GFP fluorescence intensity, with excitation at 485 ± 4.5 nm and emission at 520 ± 10 nm, were measured every 30 minutes. The effects of various chemical components on P_*mdtA*_ activity were determined at maximum fluorescence intensity. Each of the follwing was added to an appropriate final concentration, as follows: 25 μg.ml^-1^ *S*. *frugiperda* cecropin B, 25 μg.ml^-1^ cecropin A (Sigma), 1 mM MgCl_2_, 0,5 mM FeSO_4_, 2 mM CuSO_4_. CuSO_4_ was added at 2 mM to the bacterial culture at mid-exponential growth phase (O.D_600_ = 0.4). The pH of the culture medium supplemented with these compounds was adjusted to that of LB medium, and we checked that cell growth was unaffected. We also investigated the effects of insect-derived molecules on P_*mdtA*_ activity by removing HOs cleanly from 20 *L*. *migratoria* larvae and grinding them in 500 μl of cold PBS, with a microtissue grinder (Ultra-turrax, IKA). The mixture was centrifuged at 13000 *g* for 5 minutes and the supernatant was collected, filtered (0.2 μm pores) and stored at– 80°C until further use. For induction tests, the HO-derived stock solution was diluted (10-fold) in fresh LB medium. For the inhibition of proteases in bacterial and insect HO cells, Complete Mini and Complete Mini EDTA-free tablets (Roche) were added to the LB medium to yield a 0.4x solution. Plasma was collected from locust larvae by centrifuging haemolymph at 13000 *g* for 5 minutes, for the elimination of circulating cells. Plasma was then filtered (0.2 μm pores) and stored at– 80°C or used directly as a medium for bacterial inoculation. Growth data were recorded in triplicate for each set of conditions and A_600nm_ and fluorescence recorded over 48 hours of incubation were averaged for the plotting of curves and histograms. Fluorescence intensities were then normalized with respect to bacterial cell density, by dividing the fluorescence unit value (FU) recorded at a given time and in a specific condition by the corresponding A_600nm_, to obtain a specific fluorescence value. For data analysis, Relative Fluorescence Units (RFUs) were determined by the ratio of the P_*mdtA*_-*gfp*[AAV] specific fluorescence value to that of the P_*lac*_-*gfp*[AAV] in a given condition and at maximum fluorescence intensity.

### RNA preparation and quantitative RT-PCR analysis

Total RNA was extracted from *P*. *luminescens* TT01 wild-type strain cultured in LB broth (at an optical density of 0.4, corresponding to the middle of the exponential growth phase) in TRIzol reagent, according to the manufacturer’s instructions (Invitrogen). Total RNA was then purified with the High Pure RNA Isolation kit (Roche), including incubation with DNase I. RNA concentration was determined by measuring absorbance at 260 nm. For each RNA preparation, we assessed DNA contamination by carrying out a control PCR before RT-qPCR analysis.

RT-qPCR was performed in two steps. First, cDNAs were synthesised from 1 μg of total RNA with the Super Script II Reverse Transcriptase from Invitrogen and random hexamers (100 ng.μl ^-1^) from Roche Diagnostics. Quantitative PCR was then performed, in triplicate, with the Light Cycler 480 SYBR Green I Master kit from Roche Diagnostics, with 1 μl of cDNA synthesis mixture and 1 μM of specific gene primers for *mdtA* (5’-GTTTTACAAGCTCAGAAAACGAATC-3’ and 5’-ACTGATTTGGGAATAAGATGCTTTC-3’) or 16S rRNA, used as the control gene (5’- AATGGCATCTAAGACTGGTTGACTG -3’ and 5’- TACCAGGGTATCTAATCCTGTTTGC -3’). The enzyme was activated by heating for 10 minutes at 95°C. The reaction mixture was then subjected to 45 cycles of 95°C for 5 s, 60°C for 5 s and 72°C for 10 s, with the Light Cycler 480 system (Roche) used for monitoring. Melting curves were analysed for each reaction, and each curve contained a single peak. We determined the amounts of PCR products generated from standard curves obtained for PCR with serially diluted genomic DNA from *P*. *luminescens* TT01. Data were analyzed as a ratio, with 16S rRNA gene used as the control gene (95% confidence limits).

## Results

### Identification and genomic environment of the *mdtABC* operon in *Photorhabdus*

**Fig 1:**
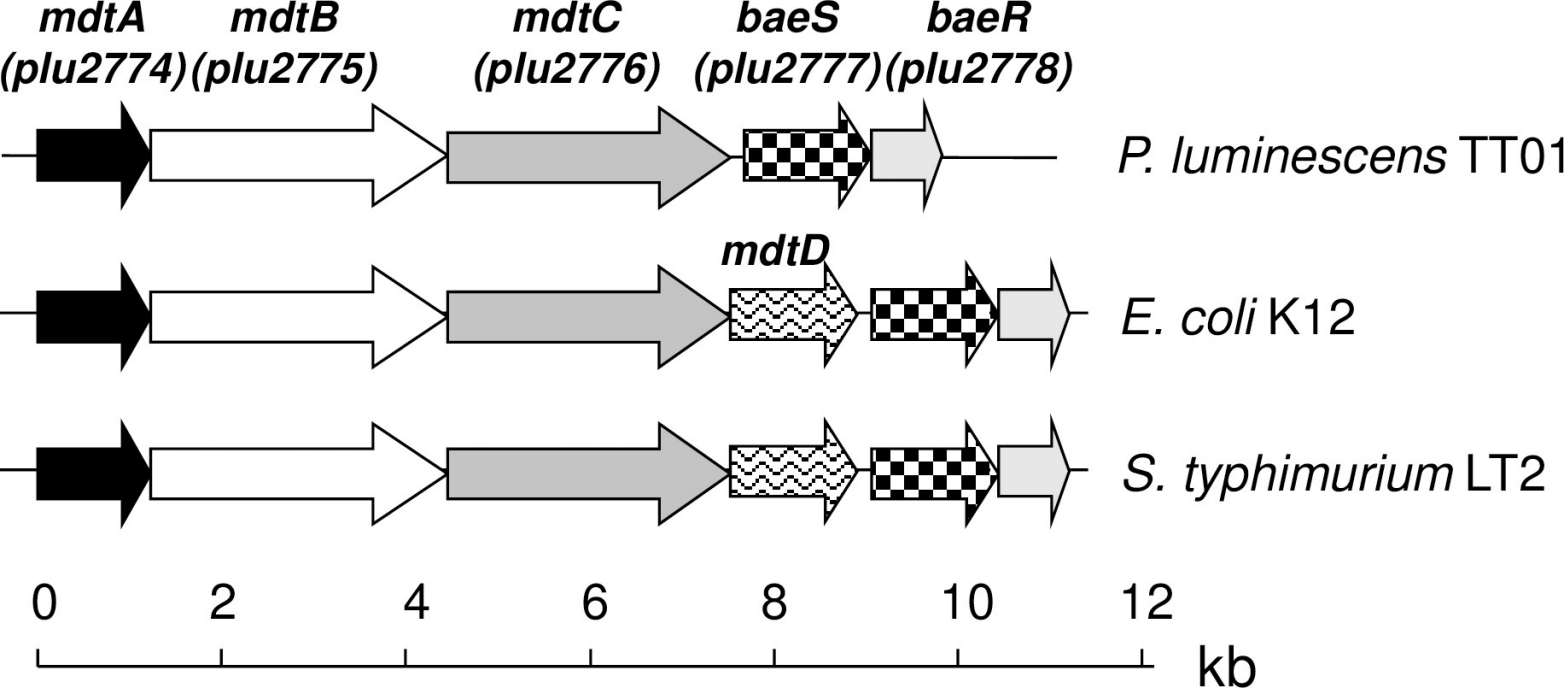
Genomic organisation of the *mdtABC* and *baeSR* loci in *Photorhabdus luminescens* TT01 and several enterobacteria. *Photorhabdus luminescens* TT01 (BX470251), *Salmonella typhimurium* LT2 (AE006468), and *Escherichia coli* K-12 (U00096). kb, kilobases.

Analysis of the *P*. *luminescens* TT01 genome sequence [47] revealed the presence of a *mdt* locus containing three open reading frames, *mdtA*, *mdtB* and *mdtC* (*plu2774* to *plu2776*) (Fig 1). The *mdtA* gene encodes a 401-amino acid (aa) putative membrane fusion protein (MFP) 64.29% (E-value of 1e^-166^) identical to the MdtA (YegM) protein of *E*. *coli*that serves as periplasmic “adaptor” connecting the inner membrane transporter (MdtB-MdtC) to an outer membrane channel such as TolC. MdtA of *P*. *luminescens* is also 81% identical to the TT01 *acrA* (*acr**iflavin* *r**esistance protein* *A* *precursor*)-encoded proteins AcrA. Both *mdtB* and *mdtC* genes encode homomultimer RND-type transporter proteins MdtB and MdtC that share 48.04% of identity and are 77.81% (E-value of 0,0) and 76.13% (E-value of 0,0) identical to MdtB (YegN) and MdtC (YegO) of *E*. *coli*, respectively. MdtB and MdtC are each partially identical to TT01 AcrB (*acr**iflavin resistance protein* *B*) (28.42% and 28.9%, respectively) and to the *plu0758* gene product (28.26% and 27.74%, respectively) which is predicted to be similar to a RND-type multidrug transporter AcrB/AcrD/AcrF with a highly conserved AcrB domain. The genome sequences of *E*. *coli* and *Salmonella* also contain a gene encoding an additional putative MFS-type drug exporter, MdtD. *mdtD* is located between the *mdtC* and *baeS* genes. However, there is no *mdtD* gene in the genome of *Photorhabdus* (Fig 1). In *E*. *coli* and *Salmonella*, the function of the MdtABC transporter requires the outer membrane channel TolC that is also found in the *Photorhabdus* genome (*plu3954*). According to Joshi and Xu (2007) in their measure of functional similarity based on Gene Ontology annotation, if two proteins have sequence identity more than 70%, they have about 90% probability or more to share the same biological process [48]. In line with this and in light of the high amino acid sequence similarity of Mdt proteins from *P*. *luminescens* and *E*. *coli*, as well as the remarkable similarities in genomic organization of the *mdtABC* operon among enterobacteria, the MdtABC complex of *P*. *luminescens* is likely to be an export system whose function also requires TolC. Moreover, the 121 nucleotides downstream from the TT01 *mdtC* stop codon constitute the *baeS/baeR* genes, which are thought to encode putative sensor kinase BaeS and response regulator BaeR, which are 61.09% and 70.74% identical to BaeS and BaeR of *E*. *coli*, respectively (Fig 1). The presence of short intergenic sequences, an absence of putative terminators between the genes and the presence of a putative terminator downstream from *baeR* strongly suggest that the *baeSR* operon may be transcribed with *mdtABC* as a monocistronic RNA, as in *E*. *coli* and *Salmonella* [28, 34].

### Phenotypic characterisation of the isogenic *P*. *luminescens* Δ*mdtA* mutant

We investigated the role of the *mdtABC* operon in *P*. *luminescens* TT01 by inactivating the *mdtA* gene by allelic exchange. The phenotypic traits of the resulting Δ*mdtA* mutant and the wild-type strain were then compared. The Δ*mdtA* and wild-type TT01 grew similarly in LB medium at 28°C, indicating that the *mdtA* mutation did not affect bacterial growth. We then assessed the role of the *mdtABC* operon in the resistance of *P*. *luminescens* to various antimicrobial compounds (bile salts, antibiotics, AMPs, detergents and metals), by determining MICs for growth for the wild-type and Δ*mdtA* strains (Table 2). The Δ*mdtA* and wild-type strains had similar susceptibilities to all the antimicrobial compounds tested. RT-qPCR experiments showed that *mdtABC* expression was ten times stronger in the complemented strain (Δ*mdtA*/pBB-*mdtABC)* but no increase in MIC was observed. Thus, *mdtABC* inactivation is not sufficient to reduce the metal or antimicrobial resistance of *P*. *luminescens*.

**Table 2:** Minimum growth-inhibitory concentrations (MICs)^*a*^ of various antimicrobial and toxic compounds for wild-type *P*. *luminescens* TT01 (TT01 WT), the Δ*mdtA* mutant and its derivatives harbouring pBBR1-MCS5 or pBB*-mdtABC*.

Antimicrobial compounds		MICs ^a^ for *Photorhabdus* strains TT01 WT, Δ*mdtA*, Δ*mdtA/*pBBR1-MCS5, Δ*mdtA/*pBB*-mdtABC*
**Bile-salts** **(**μ**g.ml**^**-1**^**)**	**DOC**	> 83000
**Detergents/** **Antiseptics** **(**μ**g.ml**^**-1**^**)**	**SDS**	312
	**TTC**	625
**Antibiotics** **(**μ**g.ml**^**-1**^**)**	**Nov**	4
	**Kan**	8
	**Ery**	97
	**Tet**	1.5
	**Amp**	31
	**Fox**	31
	**Nal**	0.6
	**Rif**	1.2
	**Cip**	0.2
	**Enr**	0.052
	**Cxm**	0.97
	**Cec**	190
	**Cro**	1250
**AMPs** **(**μ**g.ml**^**-1**^**)**	**PmB**	> 125
	**Cst**	> 500
	**Cecropin A**	> 25
	***Sf* Cecropin B**	> 125
**Metals** **(mM)**	**CuSO**_**4**_	4
	**ZnSO**_**4**_	2
	**MgSO**_**4**_	2000
**Dyes** **(**μ**g.ml**^**-1**^**)**	**BTB**	> 125
**Glycerol (%)**		25
**Flavonoids**	**Quercetin**	25
	**Saponin**	12500
	**Rutin**	>3125
**Indole (**μ**g.ml**^**-1**^**)**		97.65

^*a*^ As determined by the broth dilution method (three replicates). MICs were scored after 48 hours of incubation at 28°C. Standard deviations were equal to 0.

We also investigated the effect of efflux pump inhibitors (EPI) on both wild-type TT01 and Δ*mdtA* strain resistance to some antimicrobial agents. Phenyl-arginine-β-naphthylamide (PAβN) acts as broad-spectrum EPI in various Gram-negative bacteria [49], whereas arylpiperidines, such as 1-(1-naphthylmethyl)-piperazine (NMP) moderately decrease MDR in *E*. *coli* strains overexpressing RND-type efflux pumps [50, 51]. Both PAβN and the NMP EPIs block RND-type efflux pumps, such as *E*. *coli* AcrAB, specifically. We established the EPI concentrations to be used in assays with antimicrobial compounds at concentrations of 50 μg.ml^-1^ for NMP and 25 μg.ml^-1^ for PAβN, values one quarter and one eighth the MICs of these inhibitors for wild-type TT01 and Δ*mdtA* strains, respectively. Only PAβN significantly decreased, to various degrees, the MICs in wild-type TT01 and Δ*mdtA* of SDS, rifampicin, novobiocin, nalidixic acid and copper, with respect to the control values obtained in the absence of EPIs (Table 3). This suggests that these compounds are substrates of RND-type efflux transporters, but not solely of the MdtABC efflux pump.

**Table 3:** Minimum growth-inhibitory concentrations (MICs)^*a*^ of various antimicrobial and toxic compounds in the presence and absence of RND-type efflux pump inhibitors (PAβN or NMP) for wild-type *P*. *luminescens* TT01 (TT01 WT), the Δ*mdtA* mutant and its derivatives harbouring pBBR1-MCS5 or pBB*-mdtABC*.

Antimicrobial compounds		MICs ^a^ for *Photorhabdus* strains					
		TT01 WT			Δ*mdtA*		
		- EPIs	+ PAβN (25 μg.ml^-1^)	+ NMP (50 μg.ml^-1^)	- EPIs	+ PAβN (25 μg.ml^-1^)	+ NMP (50 μg.ml^-1^)
**Metals (mM)**	**CuSO**_**4**_	4	2	4	4	2	4
**Detergents (**μ**g.ml**^**-1**^**)**	**SDS**	312.5	< 4.8	ND	312.5	< 4.8	ND
**Antibiotics** **(**μ**g.ml**^**-1**^**)**	**Kan**	8	8	8	8	8	8
	**Rif**	1.2	< 0.07	ND	1.2	< 0.07	ND
	**Nov**	4	2	4	4	1	4
	**Nal**	0.6	0.15	0.3	0.6	0.15	0.3
**AMPs** **(**μ**g.ml**^**-1**^**)**	**PMB**	> 125	> 125	> 125	> 125	> 125	> 125
	**Cst**	> 500	> 500	> 500	> 500	> 500	> 500
	***Sf* CecB**	> 62.5	> 62.5	> 62.5	> 62.5	> 62.5	> 62.5

DOC, deoxycholate; Ery, erythromycin; Tet, tetracycline; Amp, ampicillin; BTB; bromothymol blue, Fox, cefoxitin; Cip; ciprofloxacin; Cxm; cefuroxime, Cec; cefaclor, Cro; ceftriaxone, Enr; enrofloxacin, TTC; Triphenyl tetrazolium chloride, MgSO_4_, magnesium sulphate, ZnSO_4,_ zinc sulphate; CuSO_4_, copper sulphate; SDS, sodium dodecyl sulphate; KAN, kanamycin; RIF, rifampicin; Nov, novobiocin; Nal, nalidixic acid; PmB, polymyxin B; Cst, colistin; *Sf* Cecropin B, cecropin B from *Spodoptera frugiperda*; AMPs, antimicrobial peptides; EPIs, efflux pump inhibitors; NMP, 1-(1-naphthylmethyl)-piperazine; PAβN, Phe-Arg-β-naphthylamide dihydrochloride; ND, not determined.

^*a*^ As determined by the broth dilution method (three replicates). MICs were scored after 48 hours of incubation at 28°C. Standard deviations were equal to 0.

**Fig 2:**
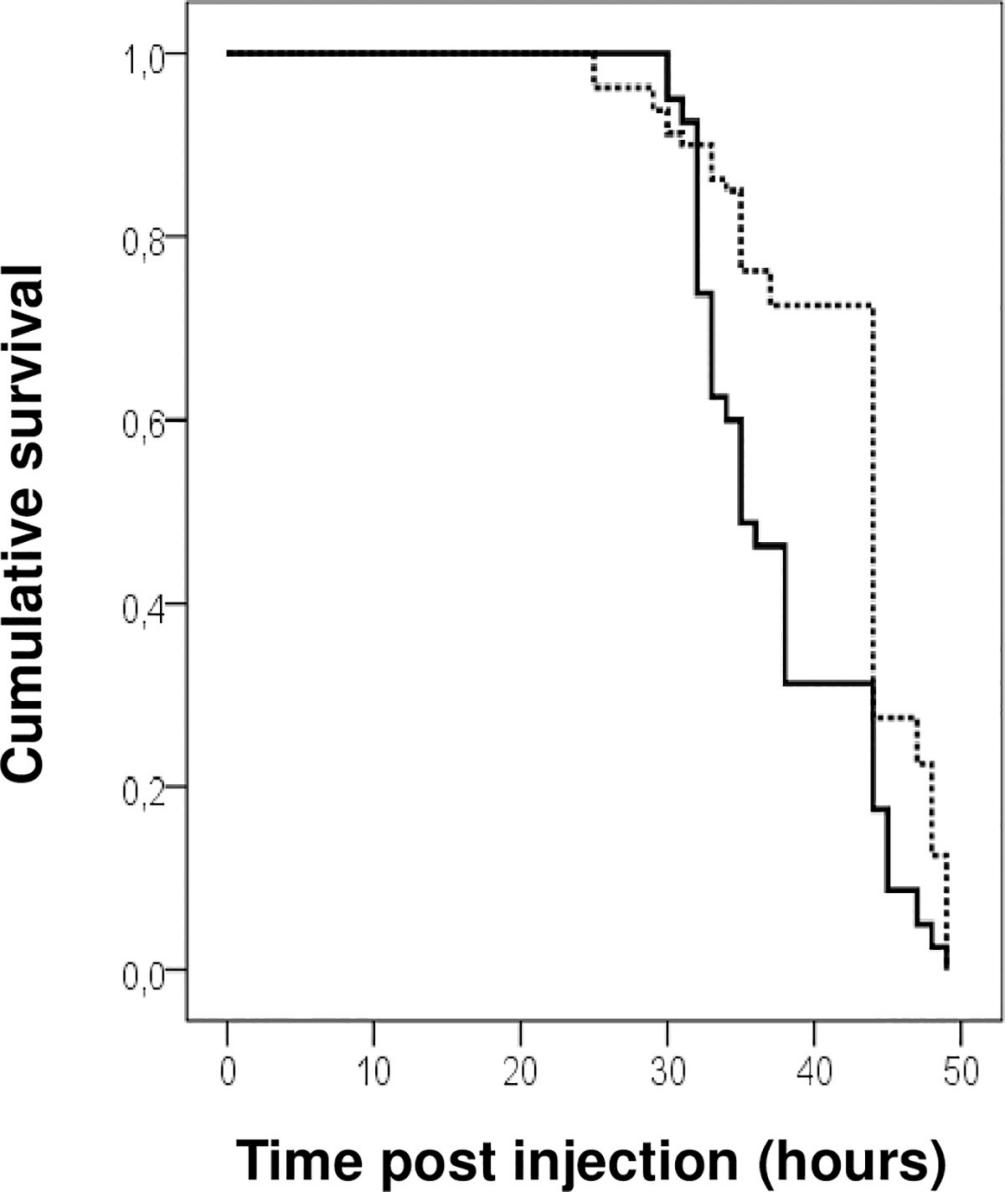
**Survival plot of *Spodoptera littoralis* insect larvae after the injection of *Photorhabdus luminescens* TT01 wild-type (black line) and *mdtA* mutant (dotted line).** Each experiment was performed with 20 larvae for each bacterium tested and the results shown are the survival functions obtained in four independent experiments. Statistical analyses with SPSS showed that the survival curve of the *mdtA* mutant was significantly different from that of the wild type (*P* < 0.0001). The calculated median survival times of larvae infected with WT and *mdtA* mutant were 34.9 h and 43.5 h, respectively.

We also investigated the role of the *mdtABC* operon in the pathogenicity of *P*. *luminescens* in insect larvae by injecting similar doses of the wild-type TT01 strain or its isogenic *mdtA* mutant directly into the haemocoel of each *Spodoptera littoralis*, *S*. *frugiperda* and *Locusta migratoria* larva, and by monitoring the insect mortality over time after the injection. Surprisingly, only bacterial injections into *S*. *littoralis* larvae showed the *mdtA* mutant to be slightly attenuated for virulence (Fig 2). These larvae infected with *mdtA* mutant cells died about 9 h later than larvae infected with the *P*. *luminescens* wild-type strain. The *mdtA* mutation had no effect on insect survival in another lepidopteran, *S*. *frugiperda*, or the locust.

### Copper increases *P*. *luminescens mdtABC* promoter activity *in vitro*

**Fig 3:**
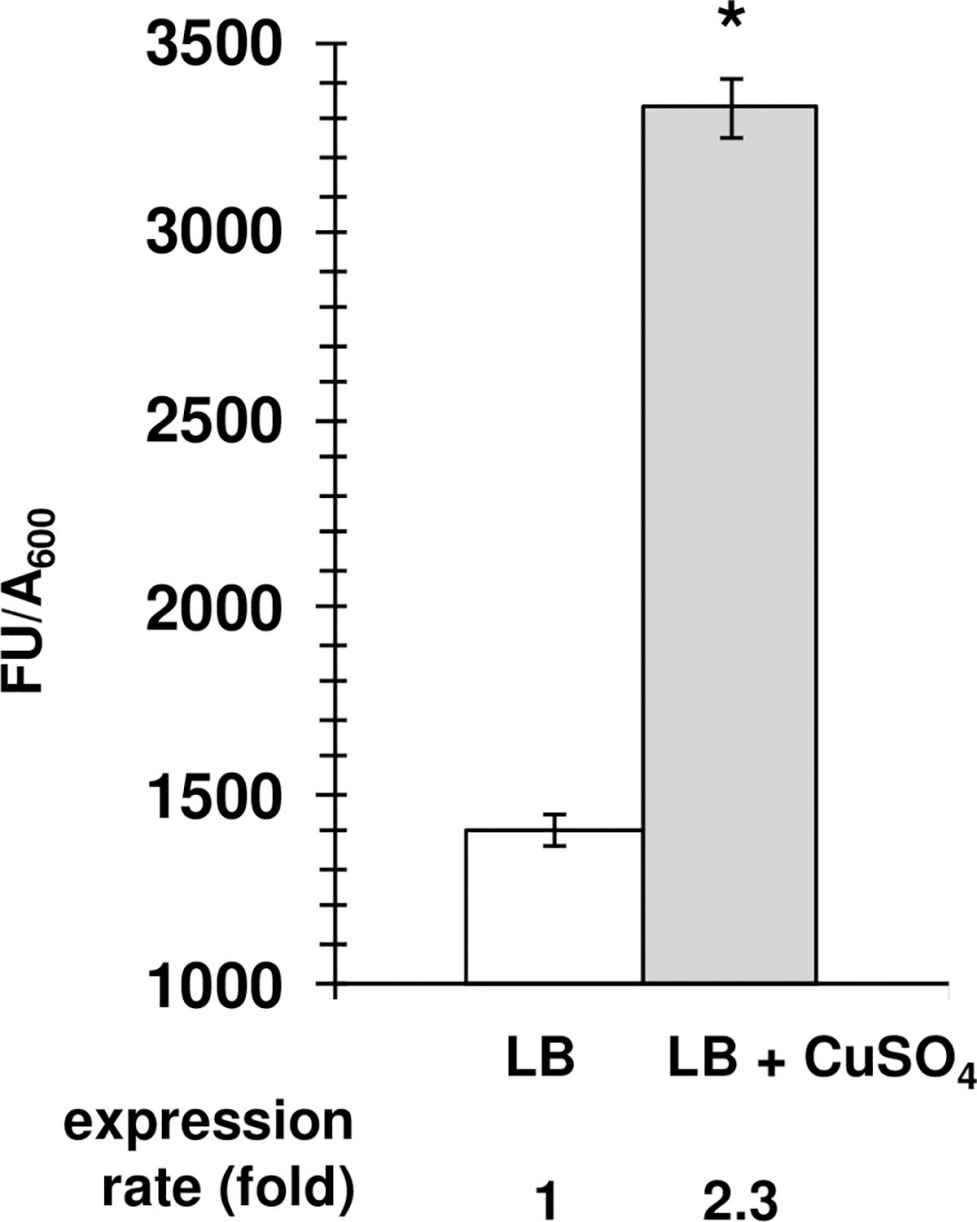
Copper significantly enhances the P_*mdtA*_*-gfp*[AAV] activity *in vitro* in *Photorhabdus luminescens*. Overnight cultures of *P*. *luminescens* carrying the P_*mdtA*_-*gfp*[AAV] fusion were diluted (1/500) in fresh LB medium in black-sided clear-bottomed 96-well plates (Greiner) and incubated at 28°C, with shaking, in a microplate reader system (TECAN). 2mM Copper was added to the bacterial culture at mid log phase (O.D_600_ equivalent to 0.4). Changes in A_600nm_ and GFP fluorescence were monitored after copper supplementation and the data shown are maximum specific fluorescence obtained by FU/A_600nm_ ratio value, calculated at six hours after the addition of copper. The results shown are the means of three experiments and asterisks indicate statistically significant differences (*, *P* < 0.05) in paired Student’s *t* tests.

For characterisation of the expression of the *P*. *luminescens mdtABC* operon, we fused *P*. *luminescens* P_*mdtA*_ to the reporter gene *gfp*[AAV] (which encodes a destabilized GFP with a half-life of ~60 min in *E*. *coli*) of pPROBE’-*gfp*[AAV] [46], a highly stable plasmid maintained without selection pressure in a sister taxon of *P*. *luminescens*, *Xenorhabdus nematophila* [52]. The expression of *mdtABC* in *Salmonella* is responsive to metals [34]. We first assessed the role of metals, such as copper, magnesium and iron, in regulating *mdtABC* gene expression in *P*. *luminescens*, by adding these metals to bacterial exponential growth-phase cultures, as described by Nishino *et al*. (2007). Similarly, we investigated the effects of AMPs from insects, such as cecropin A and *Sf* cecropin B. Only the addition of copper (2 mM) to a mid-exponential growth phase culture of *P*. *luminescens-*P_*mdtA*_-*gfp*[AAV] significantly enhanced fluorescence emission without affecting bacterial growth. At the maximum rate, recorded six hours after the addition of copper, specific fluorescence was 2.3 times higher than that in the control LB culture to which no copper was added (Fig 3).

### The *mdtABC* operon is expressed within specific insect tissues and induced *ex vivo* by insect haematopoietic organ extracts

**Fig 4:**
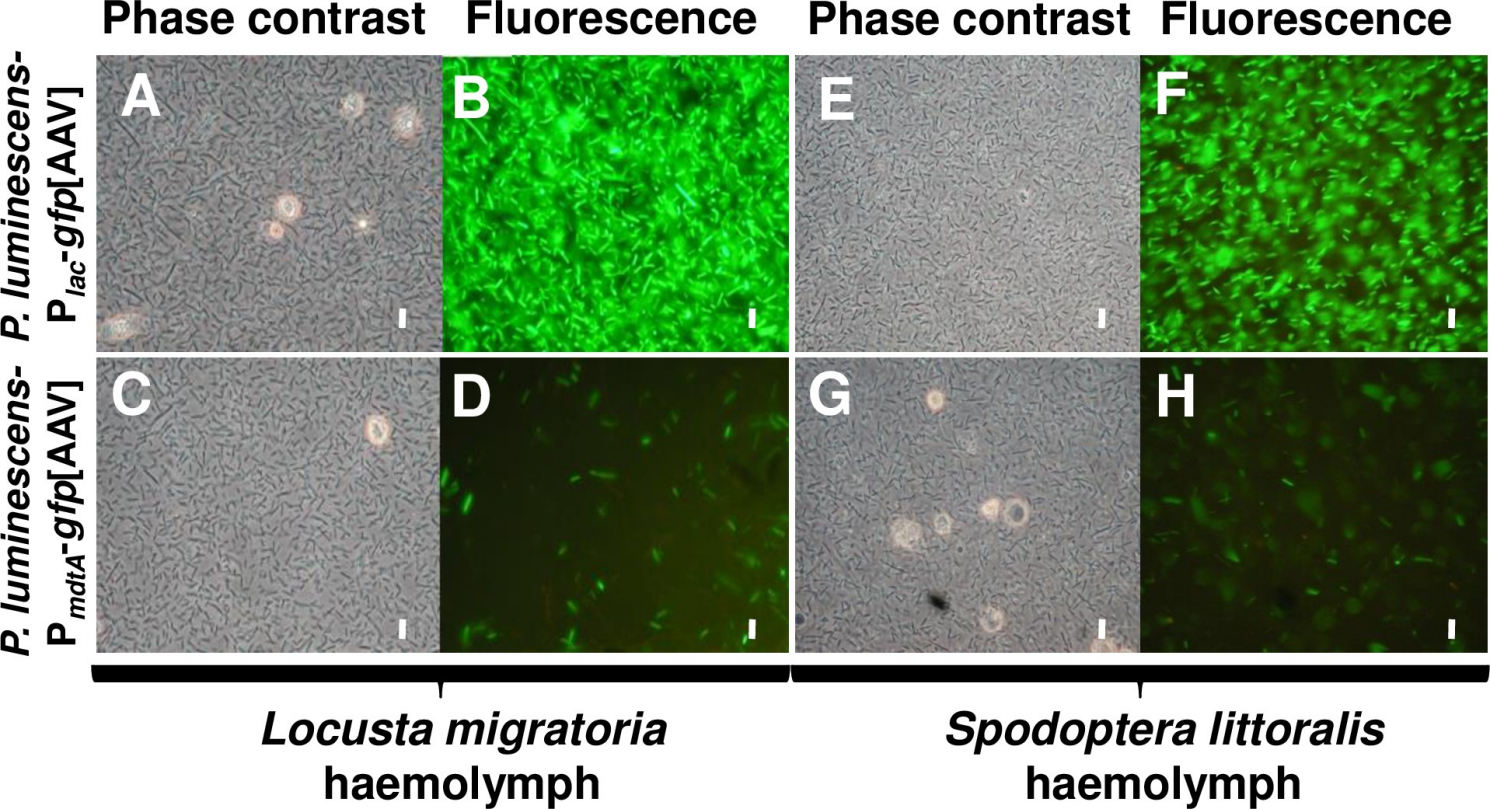
Comparison of the *in vivo* expression of the *Photorhabdus luminescens mdtABC* operon and constitutive GFP expression in *Locusta migratoria* and *Spodoptera littoralis* haemolymphs. Insects were injected with wild-type *P*. *luminescens* harbouring a transcriptional fusion between *E*. *coli P*_*lac*_ (A, B, E, and F) or the *mdtABC* (C, D, G, and H) promoter and the *gfp*[AAV] reporter gene. Haemolymph was regularly extracted from infected larvae during infection, observed under a light microscope (A, C, E, and G) and fluorescence was determined (B, D, F, and H). The observations shown were made at 20 to 24 hours post-injection for *S*. *littoralis* larvae and at 30 hours post-injection for *L*. *migratoria*, and correspond to the results of at least three independent experiments. Scale bar represents 10 μm.

**Fig 5:**
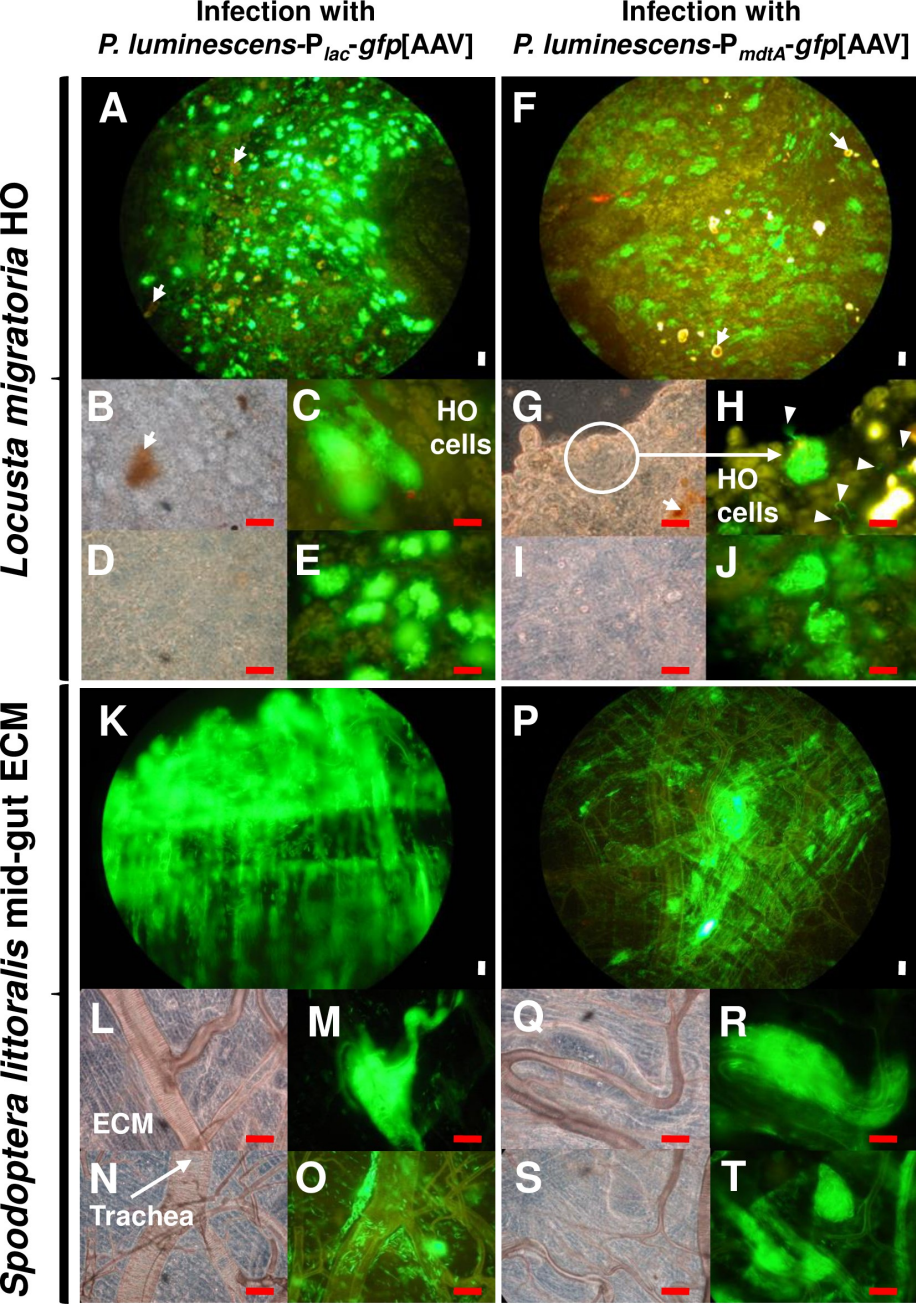
*In vivo* site-specific expression of the *Phototrhabdus luminescens mdtABC* operon during colonization of *Locusta migratoria* HO and *Spodoptera littoralis* midgut. Insects were injected with recombinant *P*. *luminescens-*P_*lac*_*-gfp*[AAV] (A-E and K-O) or with recombinant *P*. *luminescens-*P_*mdtA*_*-gfp*[AAV] (F-J and P-T). HO tissues were observed under a light microscope (B, D, G, I; L, N, Q, and S), or by fluorescence microscopy (A, C, E, F, H, and J). Nodules formed after the injection of *P*. *luminescens* TT01 or its derivatives into the *L*. *migratoria* haemocoel are indicated with white arrows (A, F, B, and G). Note the presence of bacterial aggregates within the connective tissues of the HO in *L*. *migratoria* (A and F) and in the vicinity of the midgut in *S*. *littoralis* (K and P), each injected with *Photorhabdus*. Pieces of HO in which nodule structures were visible (B and G) or not visible (D and I) were observed under the epifluorescence microscope (C and E, H and J, respectively). Brightly fluorescent green bacteria were detected in both cases, demonstrating the HO site-specific expression of P_*mdtA*_*-gfp*[AAV] within closed bacterial aggregates, independently of nodule structures. The circle (G) indicates the limit of a bacterial aggregate and arrowheads (H) indicate isolated bacteria. All these observations were made 20 to 28 hours post-injection for *S*. *littoralis* and at 30 hours post-injection for *L*. *migratoria*, and correspond to results of at least three independent experiments. ECM, extracellular matrix. Red scale bar represents 10 μm. White scale bar represents 50 μm.

For the characterisation of *mdtA* expression *in vivo*, we injected the TT01 strain carrying the P_*mdtA*_*-gfp*[AAV] fusion or the P_*lac*_**–***gfp*[AAV] fusion (positive control) into the haemocoel of *L*. *migratoria* last-instar larvae (10^4^ bacteria/larva) and *S*. *littoralis* fifth-instar larvae (10^3^ bacteria/larva). We then monitored the presence of bacteria for up to 30 hours after infection in haemolymph, the locust haematopoietic organ (HO), and *S*. *littoralis* mid-gut connective tissues. We first localized *P*. *luminescens-*P_*lac*_-*gfp*[AAV] fluorescent bacteria to the sites of preferential colonization: the haemolymph (Fig 4A, 4B, 4E and 4F), the HO of *L*. *migratoria* (Fig 5A), and the mid-gut connective tissues of *S*. *littoralis* (Fig 5K) from 20 hours to 30 hours after infection.

After injection of the *P*. *luminescens-*P_*mdtA*_-*gfp*[AAV] strain, only 5% of fluorescent bacteria were detected after recovery from haemolymph at 20 to 24 hours (*S*. *littoralis*) and 30 hours post-injection (*L*. *migratoria*) (Fig 4D and 4H). However, All *P*. *luminescens*-P_*lac*_-*gfp*[AAV] recovered from the haemolymph at the same time points were fluorescent (Fig 4B and 4F). Thus, P_*mdtA*_ activity is almost entirely absent from the haemolymph in both *Locusta* and *Spodoptera*. In *Locusta*, numerous nodules were observed in the HO after the injection of *P*. *luminescens-*P_*lac*_-*gfp*[AAV] (Fig 5A and 5B). Constitutively fluorescent *P*. *luminescens* bacteria colonised the connective tissues of the locust HO and the lepidopteran mid-gut as bacterial aggregates, as observed under a fluorescence microscope (Fig 5A, 5C, 5E, 5F, 5H and 5J). The localisation of bacterial aggregates was not necessarily associated with the presence of nodules (Fig 5B–5E). Moreover, fluorescence microscopy observations of HO and mid-gut connective tissues showed that bacteria harbouring the P_*mdtA*_-*gfp*[AAV] fusion were fluorescent within aggregates (Fig 5F, 5H and 5J). Not all the fluorescent bacteria observed were in contact with nodules and the trachea in *Locusta* HO and *Spodoptera*, respectively, suggesting that the P_*mdtA*_ activity was not related to these structures (Fig 5I, 5J, 5S and 5T).

**Fig 6:**
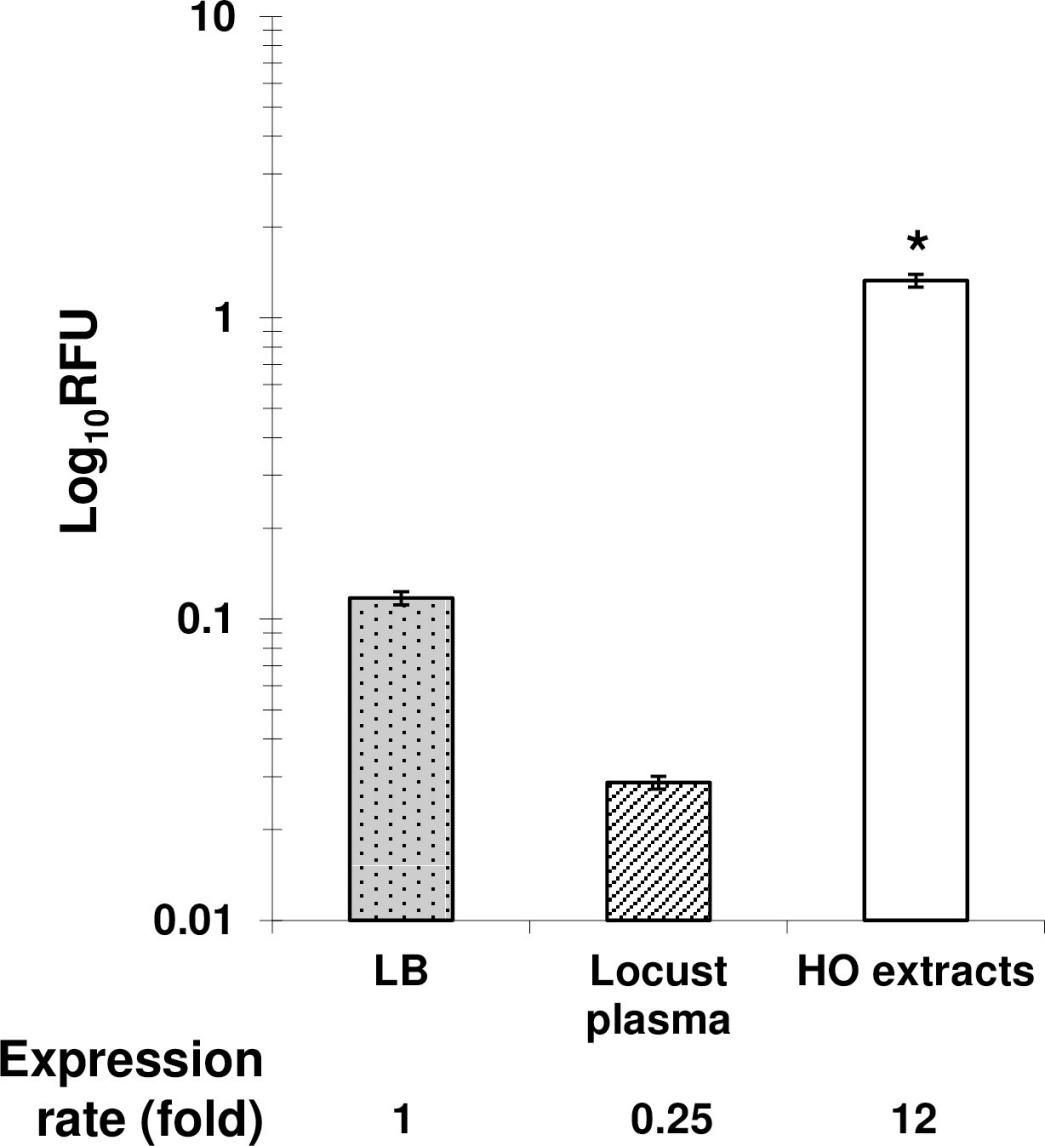
Level of expression of *Photorhabdus luminescens mdtABC* operon in *Locusta migratoria* plasma and medium containing HO extract *in vitro*. Overnight cultures of *P*. *luminescens* carrying the P_*mdtA*_*-gfp*[AAV] fusion were diluted (1/500) in black-sided clear-bottomed 96-well plates containing one of the following media: fresh LB medium, plasma or fresh LB medium supplemented with *L*. *migratoria* HO extract. A_600nm_ and GFP fluorescence were monitored, in real time, in triplicate for each set of conditions, at 28°C, with orbital shaking, in an Infinite M200 microplate reader (TECAN). Specific fluorescence was obtained by dividing GFP fluorescence units (FU) by the A_600nm_ value at 20 hours of growth. Relative Fluorescence Units (RFUs) were determined by the ratio of the P_*mdtA*_-*gfp*[AAV] specific fluorescence value to that of the P_*lac*_-*gfp*[AAV]. Asterisks indicate statistically significant differences (*, *P* <0.05) in the paired Student’s *t* test. The expression rate is the ratio between specific activity obtained after incubation with HO extracts and that obtained after incubation with insect plasma.

For further confirmation of MdtABC induction *ex vivo*, we monitored the fluorescence of *P*. *luminescens*-P_*mdtA*_-*gfp*[AAV] grown in the plasma (cell-free haemolymph) of locust larvae or in HO homogenate-containing medium. We found that P_*mdtA*_ activity was almost entirely absent when bacteria were grown in plasma, but very strong in locust HO extracts (Fig 6). Indeed, the specific fluorescence of *P*. *luminescens*-P_*mdtA*_-*gfp*[AAV] in the presence of locust HO extracts measured *in vitro* was at least 12 times stronger than that recorded in the plasma of the same insect (Fig 6). These experiments clearly support the *in vivo* observations and indicate that the P_*mdtA*_ activity is tissue-specific. Moreover, we observed under fluorescence microscopy bacteria from culture-based induction assays at different time intervals and during peak expression. We first showed that both planktonic and aggregated bacterial cells from HO-containing cultures strongly expressed GFP. However, bacteria grown in either plasma or LB showed slight expression similar to what we have observed in the plasma from infected insects. These results suggestthat *mdtABC* expression in locust HO was unrelated to aggregate formation. Such a transient (late stages of infection) and spatially (connective tissues) specific expression of *mdtABC* operon most probably improve bacterial dissemination and persistence into the insect cadaver. Moreover, giving the strong activity of the P_*mdtA*_ reported both *ex vivo* and *in vivo* in the locust HO, we wanted to test the phenotype of *Photorhabdus* under these conditions regarding MICs changes *in vitro*. *P*. *luminescens mdtA* mutant and wild-type strains exhibited the same MICs for SDS, Kanamycin, copper, and novobiocin, in the presence or absence of HO extracts (S1 Table).

### HO-mediated *mdtABC* expression is reduced by protease inhibitors

For preliminary characterisation of the HO-derived putative signals for *mdtABC* expression, the TT01 strain carrying the P_*mdtA*_*-gfp*[AAV] fusion in LB supplemented was grown with HO extracts untreated or previously heated at 60°C. The use of heated HO extracts resulted in significantly lower levels of P_*mdtA*_*-gfp*[AAV] activity (S1 Fig), indicating that the stimuli upregulating *mdtABC* expression are non-heat stable and could be proteinaceous in nature (proteins or peptides).

**Fig 7:**
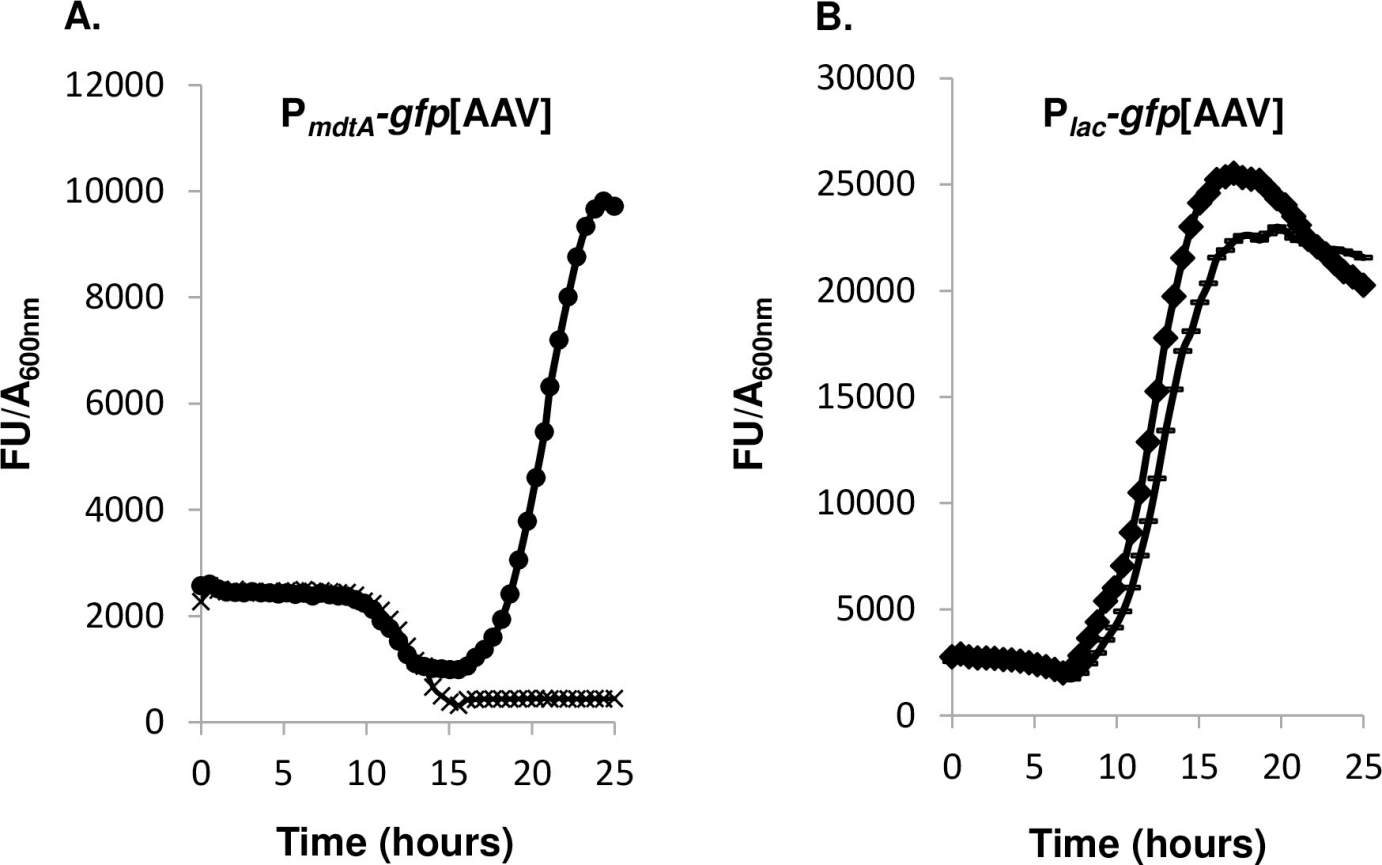
Cysteine and serine protease inhibitors prevent expression of the *mdtABC* operon. TT01 strains carrying the P_*mdtA*_*–gfp*[AAV] (A) or P_*lac*_*–gfp*[AAV] (B) fusions were grown in LB medium containing HO extracts and supplemented (**×** and □) or not supplemented (● and ♦) with complete mini EDTA-free protease inhibitor. Specific fluorescence is expressed as the ratio of GFP fluorescence units (FU) to A_600nm_ value. The results are representative of three independent assays.

*Photorhabdus* produces high numbers of proteases during the colonization of insect host tissues [5]. We therefore focused on the protease-dependent modulation of *mdtABC* gene expression. We monitored the expression of both P_*mdtA*_*-gfp*[AAV] and P_*lac*_*-gfp*[AAV] fusions in TT01 strain, by recording GFP fluorescence in LB supplemented with HO extracts in the presence and absence of complete mini EDTA-free protease inhibitor, which fully inhibits a broad spectrum of serine and cysteine proteases (Fig 7A). We first showed that a 0.4x solution of EDTA-free protease inhibitor had no effect on bacterial growth (data not shown). We then showed that expression of the P_*mdtA*_*-gfp*[AAV] fusion was inhibited by the addition of EDTA-free protease inhibitor to the culture medium containing the inductive HO extract, no such inhibition being observed with non-supplemented control medium (Fig 7A). As a control, expression of the P_*lac*_*-gfp*[AAV] fusion remained very strong in the presence of HO extracts and inhibitors (Fig 7B). Thus, expression of the *mdtABC* operon in *Photorhabdus* is dependent on proteolysis by-products.

## Discussion

We examined the role of the putative MdtABC efflux pump in *P*. *luminescens* MDR and virulence. *P*. *luminescens* Δ*mdtA* was as resistant as the wild-type strain to common substrates of multidrug transporters, such as DOC, SDS and novobiocin. We therefore suggest that the MdtABC multidrug transporter alone is not required for MDR in *P*. *luminescen*s, at least *in vitro*. However, this is not surprising because the deletion of single RND drug efflux pump genes, including the *mdtABC* operon of *S*. *enterica* and *E*. *coli*, has no significant effect on drug resistance, other than in *acrAB* mutants [25, 34]. As mentioned in results section, other putative RND pumps-encoding genes are present in the genome of *P*. *luminescens* TT01 such as *acrAB* and *acrB*/*acrD*/*acrF* homologues, that probably contribute to MDR resistance in *Photorhabdus* as described in other enterobacteria [22, 25, 53, 54]. This hypothesis is strongly supported by our findings that RND inhibitors significantly enhanced bacterial susceptibility to SDS, rifampicin, novobiocin and nalidixic acid, all of which are common substrates of RND efflux pumps. Although some substrates of the MdtABC efflux pump were characterized in some bacteria, most of its known substrates, such as antibiotics and detergents, are not encountered in the natural environment of these bacteria. In this study, we showed that the genetic control of the MdtABC-mediated efflux is characterized by a well regulated expression of the *mdtABC* genes in specific spatial (connective tissues) and temporal (late infection stages) patterns. Therefore, it seems likely that the MdtABC efflux system may have evolved for specific purposes during the interaction of *Photorhabdus* with its invertebrate hosts from symbiosis with nematodes to pathogenicity for insects. In this regard, it is noteworthy that the natural environment of *Photorhabdus*, especially the insect cadaver, is enriched in antimicrobial compounds produced by *Photorhabdus* itself, such as potentially toxic metabolites [55–57], bacteriocins [58], and stilbene [59–61], as well as other antibiotics that are released by the competitive microbiota of both the nematode and the insect [62, 63].

Bacterial multidrug efflux pumps of the RND family have been shown to be important for virulence in model animals, following infection via several routes, including ingestion, inhalation and the intraperitoneal injection of bacteria [64, 65]. We show here that *P*. *luminescens* Δ*mdtA* is as virulent as the wild-type strain after injection into haemocoel of the locust and the lepidopterans *Spodoptera*. These findings were expected given that *Photorhabdus* has a plenty of different and redundant virulence factors ranging from potent toxins and enzymes to PhoP-regulated LPS modification systems mediating resistance to AMPs produced in insect haemolymph upon infection [9]. Therefore, it is often hard to identify an effect on insect pathogenicity when just a single gene or operon that might contribute to virulence is inactivated. The only genes currently described as essential for *P*. *luminescens* resistance to AMPs and virulence in insects are *phoP* and *pbgPE* [66, 67], that were recently shown to govern the main virulence strategy of an AMP resistant subpopulation of *P*. *luminescens* causing septicemia in insects [68]. Previous studies of the colonization of insect larvae by *P*. *luminescens* have shown that the extracellular matrix of the connective tissues and haemolymph are initially more extensively colonized than other tissues [4, 5]. We showed, by transcriptional fusion with the *gfp* gene, that the P_*mdtA*_ activity of *Photorhabdus mdtABC* operon was clearly increased in the connective tissues, but not in the plasma, during the infection of three insect species. We mimicked the ‘host-like’ conditions triggering the MdtABC efflux system encountered during bacterial colonization of the extracellular matrix, by dissecting the HO from *Locusta*. Only a moderately developed connective tissue enclosing the belts of muscle around the mid-gut is found in lepidopterans, such as *Spodoptera*. By contrast, a well-developed connective tissue with specialist cells and extracellular material, often organised into structured fibres (collagen), has been described in the HO along the dorsal vessel in *Locusta migratoria* [69]. Our *ex vivo* findings provide strong evidence for a specific signal within the HO but apparently absent from insect plasma. Collagen is a normal constituent of the animal extracellular matrix, including that of the insect *L*. *migratoria*, which has been shown to possess two types of collagen: a predominant fibrous collagen similar to collagen I from mammals and a minor collagenous component very similar to basement membrane collagen IV [69]. Preliminary assays showed that mammalian collagen IV and collagen I had no effect on *mdtABC* expression. Our studies have shown that expression of the *lopT* gene, encoding an antiphagocytic type three secretion system (TTSS) effector, is induced only at sites of cellular defence reactions, such as nodules in the HO of *L*. *migratoria* [4]. We show here that *mdtABC* expression is also site-specific but independent of these defence reactions, because fluorescent bacteria were not only associated with nodule formation in the HO.

Our data revealed that the inhibition of cysteine and serine proteases in HO-supplemented medium was sufficient to inhibit the *ex vivo* expression of P_*mdtA*_*-gfp*[AAV]. Indeed, *Photorhabdus* produces a wide range of proteases, including metalloproteases and non-metalloproteases, in culture media or in contact with insect tissues during infection [70–72]. PrtA, a metalloprotease inhibited by disulfide bridge-reducing agents and SH group reagents, is a good candidate that is produced within the connective tissues close to the midgut epithelium during late infection stages in insects when septicaemia is well established [73, 74]. PrtA degrades basal lamina, providing the bacteria with access to the underlying tissues for bioconversion [5]. Moreover, the genome of the TT01 strain contains other sequences potentially corresponding to cysteine and serine protease- or peptidase-encoding genes [47]. The peptides and/or amino acids generated by the proteolytic action of these bacterial proteases on the HO tissue probably trigger the production of the MdtABC pump *in vivo*.

Sensing of and responding to environmental fluctuations via two-component systems are of vital importance for bacteria [75, 76], especially in the specific context of MDR [75, 77, 78]. Indeed, the BaeSR two-component system of *E*. *coli* and *Salmonella* detects specific envelope-damaging compounds (indole, zinc, copper and certain flavonoids) and induces the MdtABC efflux pump, which can expel these inducing compounds, as well as some antibiotics and bile salt derivatives, thereby maintaining bacterial envelope homeostasis [23, 34, 35, 79, 80]. The *baeS* and *baeR* genes are found immediately downstream from *mdtABC* in *P*. *luminescens* TT01 genome, suggesting that BaeSR may also regulate the expression of the *mdt* operon. This supports the hypothesis that the *mdtA*-inducing compounds within the insect HO are sensed by the *Photorhabdus* BaeSR pathway. Our data indicate that *mdtABC* expression is modulated by copper *in vitro*, but no change in *P*. *luminescens* copper resistance was observed with the *mdtA* mutant strain. This finding further suggests that the MdtABC pump may be one of multiple redundant systems contributing to metal homeostasis in *Photorhabdus* as demonstrated for *Salmonella*, and that other transporters may function independently or in synergy with MdtABC to increase copper resistance. This hypothesis is supported by our finding that *mdtA* gene was weakly expressed in the widely used laboratory LB medium, and that such expression was slighlty increased after copper supplementation. Copper in insects may derive from phenoloxidase that is a metalloenzyme involved in nodule formation [81]. As we already showed that the P_*mdtA*_ activity was slightly increased (2.3 folds) by copper *in vitro*, we investigated whether copper induces MdtABC site-specific expression within the HO *ex vivo*. This seems consistent with the fact that proteases such as PrtA produced at later stages of infection could cause copper release from phenoloxidase in the HO, resulting in the increase in steady state levels of *mdtABC* expression. Nevertheless, we demonstrated that addition of a copper chelator to the HO extracts-containing medium did not affect GFP levels emitted by *P*. *luminescens-*P_*mdtA*_-*gfp*[AAV] comparing to a copper chelator-free medium, thereby suggesting that copper is not responsible of activating *mdtABC* expression within the HO.

The aim of this study was to elucidate the inducing cues and function of the putative MdtABC efflux pump in *P*. *luminescens*. Although no clear phenotype for the *mdtA* mutant has been identified, it is tempting to speculate that the Bae stress response pathway involving MdtABC may be involved in fostering *Photorhabdus* survival to the complex stresses of its hosts [82]. Microbes that can grow within the constraints of the insect cadaver may, themselves, produce stress through competitive or antagonistic interactions. Therefore, microbial activity in host tissues, or interactions with specific microbial taxa within the community, may induce multiple stresses that symbiotic *Photorhabdus* should overcome in order to increase their fitness and specific recruitment by the infective juvenile nematodes. These hypotheses are highly consistent with our findings concerning the site-specific expression of MdtABC during late infection stages prior to insect death. Further studies are required to determine the precise nature of signals involved in MdtABC induction as well as its natural substrates in *P*. *luminescens*. For instance, we suggest that these inducers could belong to by-products resulting from the action of proteases on HO matrix compounds. This would constitute a novel type of cross-talk, highlighting the versatility of efflux pumps in responses to the various compounds generated or encountered by bacteria in their surrounding environment.

## Supporting information

S1 Table Minimum growth-inhibitory concentrations (MICs)^*a*^ of SDS, Kanamycin, copper, and novobiocin, for wild-type *P*. *luminescens* TT01 (TT01 WT), the Δ*mdtA* mutant and its derivatives harbouring pBBR1-MCS5 or pBB*-mdtABC*, in the presence or absence of HO extracts. (PDF)

S1 Fig Heating of HO extracts abolishes the expression of the P_*mdtA*_-*gfp*[AAV] fusion *in vitro*. TT01 strain carrying the P_*mdtA*_*–gfp*[AAV] fusion was grown in LB medium containing HO extracts previously heated at 60°C (**×**) or not heated (●). Specific fluorescence is expressed as the ratio of GFP fluorescence to Absorbance at 600 nm. The results are representative of three independent assays.
(TIF)

S2 Fig Microscopic observations of locust HO and *Spodoptera* midgut ECM infected with *P*. *luminescens* harbouring a promoter less fusion pPROBE’-*gfp*[AAV]. These figures serve as negative controls to show that bright fluorescence observed in Figs 4 and 5 is specific to P_*mdtA*_*-gfp*[AAV] and P_*lac*_*-gfp*[AAV] and that is not autofluorescence of insect tissues. Insects were injected with recombinant *P*. *luminescens-* pPROBE’*-gfp*[AAV]. Insect tissues and haemolymphs were observed by fluorescence microscopy. All these observations were made 20 to 28 hours post-injection for *S*. *littoralis* and at 30 hours post-injection for *L*. *migratoria*, and correspond to results of at least three independent experiments. ECM, extracellular matrix.
(TIF)

S1 Dataset Values behind the means and standard deviations used to build graph of Fig 3. (XLS)

S2 Dataset Values behind the means and standard deviations used to build graph of Fig 6. (XLS)

S3 Dataset Values behind the means and standard deviations used to build graph of Fig 7. (XLS)
