# Supplementary material for: Spatiotemporal expression of the putative MdtABC efflux pump of Phtotorhabdus luminescens occurs in a protease-dependent manner during insect infection
Source: PLoS One. 2019 Feb 14;14(2):e0212077. doi: 10.1371/journal.pone.0212077 (PMC6375597; doi:10.1371/journal.pone.0212077)
Supplement: S1 Table — (PDF) [file pone.0212077.s001.pdf]

**Antimicrobial compounds**	**MICs [a] for *Photorhabdus* strains TT01 WT, Δ*mdtA*, Δ*mdtA*/pBBR1-MCS5, Δ*mdtA*/pBB-*mdtABC* in the presence or absence of HO-extracts**
**DOC**	> 83000 µg.ml^{-1}
**Kan**	8 µg.ml^{-1}
**Nov**	4 µg.ml^{-1}
**$CuSO_4$**	4 mM
