# Supplementary figures and images for: Spatiotemporal expression of the putative MdtABC efflux pump of Phtotorhabdus luminescens occurs in a protease-dependent manner during insect infection
Source: PLoS One. 2019 Feb 14;14(2):e0212077. doi: 10.1371/journal.pone.0212077 (PMC6375597; doi:10.1371/journal.pone.0212077)

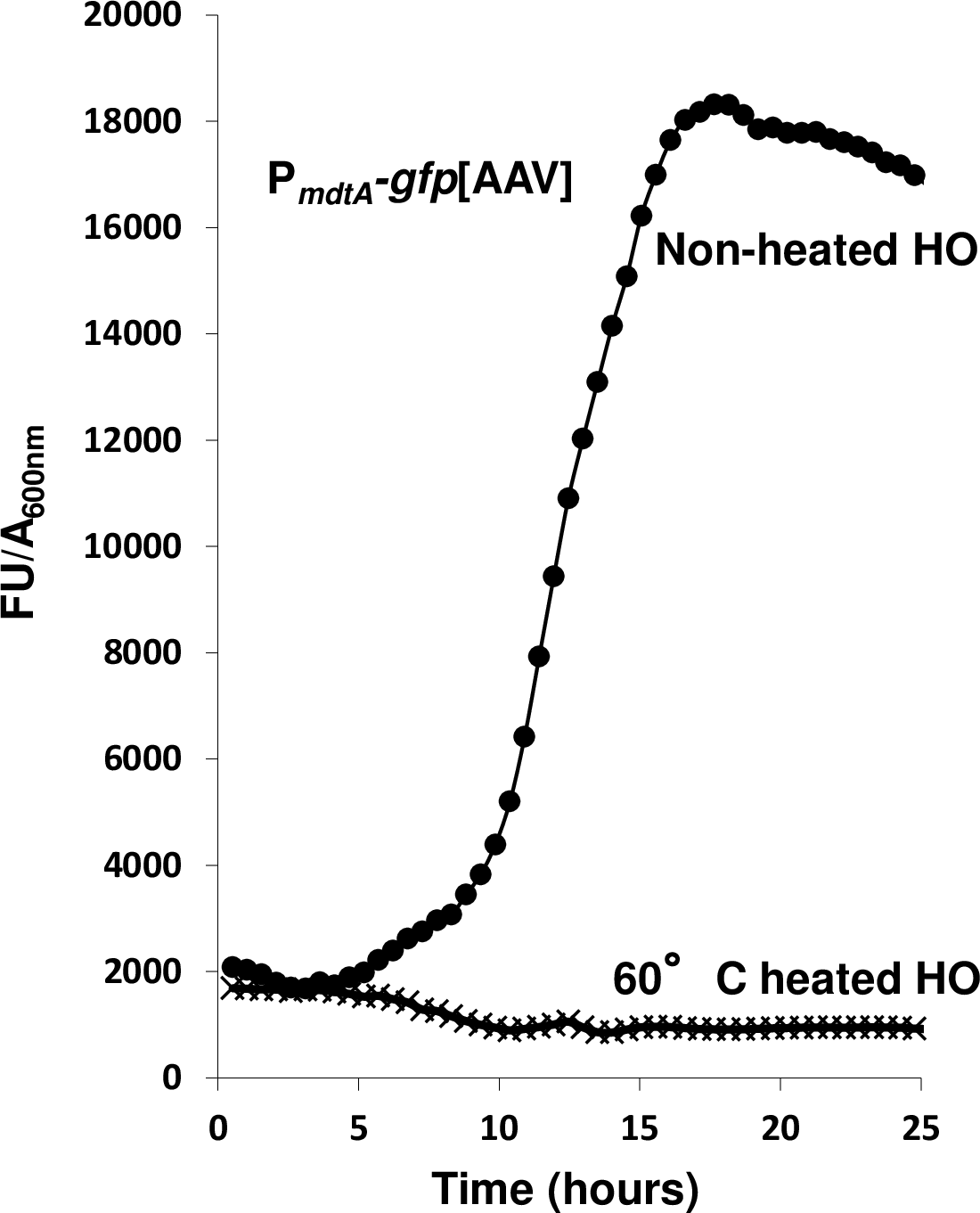

Supplement: S1 Fig — TT01 strain carrying the PmdtA–gfp[AAV] fusion was grown in LB medium containing HO extracts previously heated at 60°C (×) or not heated (●). Specific fluorescence is expressed as the ratio of GFP fluorescence to Absorbance at 600 nm. The results are representative of three independent assays. (TIF) [file pone.0212077.s002.tif]

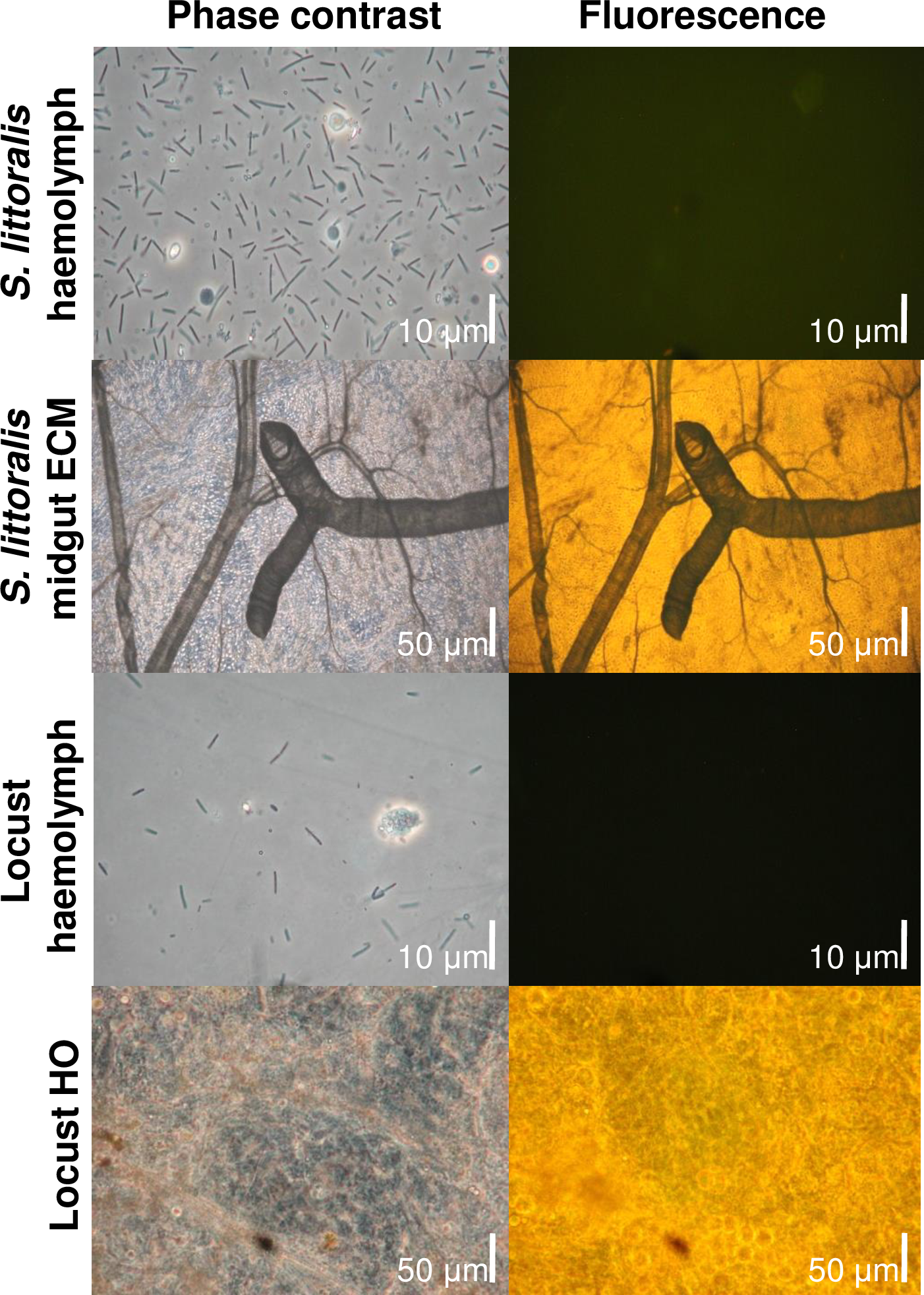

Supplement: S2 Fig — These figures serve as negative controls to show that bright fluorescence observed in Figs 4 and 5 is specific to PmdtA-gfp[AAV] and Plac-gfp[AAV] and that is not autofluorescence of insect tissues. Insects were injected with recombinant P. luminescens- pPROBE’-gfp[AAV]. Insect tissues and haemolymphs were observed by fluorescence microscopy. All these observations were made 20 to 28 hours post-injection for S. littoralis and at 30 hours post-injection for L. migratoria, and correspond to results of at least three independent experiments. ECM, extracellular matrix. (TIF) [file pone.0212077.s003.tif]
